# Maleimide index: a paleo-redox index based on fragmented fossil-chlorophylls obtained by chromic acid oxidation†

**DOI:** 10.1039/d2ra04702k

**Published:** 2022-10-31

**Authors:** Kenta Asahina, Satoshi Takahashi, Ryosuke Saito, Kunio Kaiho, Yasuhiro Oba

**Affiliations:** Research Institute for Geo-Resources and Environment, Geological Survey of Japan, National Institute of Advanced Industrial Science and Technology (AIST) Central 7, 1-1-1 Higashi Tsukuba Ibaraki 305-8567 Japan kenta-asahina@aist.go.jp +81-29-861-2391; Department of Earth and Environmental Sciences, Nagoya University Furo-cho, Chikusa-ku Nagoya Aichi 464-8601 Japan; Department of Geosphere Sciences, Yamaguchi University 1677-1 Yoshida, Yamaguchi City Yamaguchi 753-8512 Japan; Japan Science and Technology Agency PRESTO, 4-1-8 Honcho Kawaguchi Saitama 332-0012 Japan; Department of Earth Science, Tohoku University Aoba-aza, Aramaki, Aoba-ku Sendai 980-8578 Japan; Institute of Low Temperature Science, Hokkaido University N19W8, Kita-ku Sapporo Hokkaido 060-0819 Japan

## Abstract

The composition of past photosynthetic organisms provides information about the paleo-environment based on the habitat characteristics of photosynthetic organisms. Therefore, analysis of chlorophyll-derived materials from photosynthetic organisms in sedimentary rocks is important for understanding paleo-environmental changes. Fossilized chlorophylls present in sedimentary rocks can be detected by their conversion into maleimides and phthalimides. This can be achieved through the chromic acid oxidation of sedimentary rocks. Since the maleimides and phthalimides are derived from the pyrrole skeleton of fossil chlorophylls, their composition reflects the composition of paleo-photosynthetic organisms. We herein propose an indicator for detecting anoxic-sulfidic conditions in the paleo oceanic photic zone, which is based on the composition ratio of the maleimides produced during the oxidation process. The maleimide index in this study would be a useful analytical method to indicate that anoxic-sulfidic conditions in the paleo oceanic photic zone, which is associated with mass extinction events, have occurred.

## Abstract 2

In this study, a paleo-redox index using chlorophyll-derived substances incorporated into insoluble polymers in sedimentary rocks was proposed.

## Graphical abstract

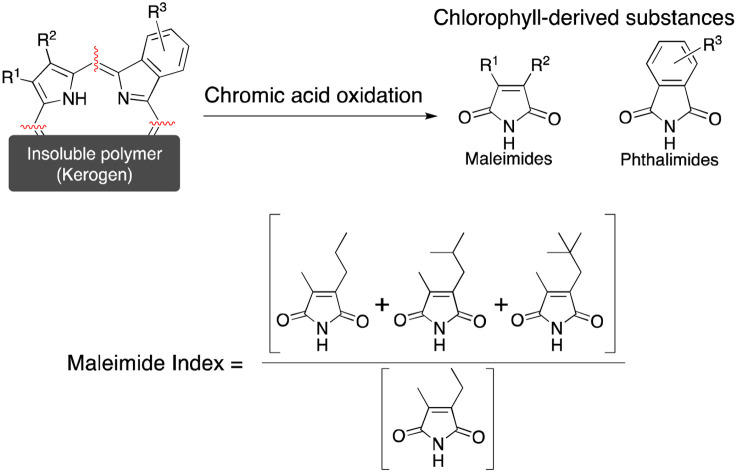

## 1 Introduction

Porphyrins and their related substances are commonly found in sedimentary rocks, crude oils, and recent sediments.^1–21^ These compounds are considered to be degradation products of the chlorophylls possessed by photosynthetic organisms that lived in the past.^1–22^

**Chart 1:**
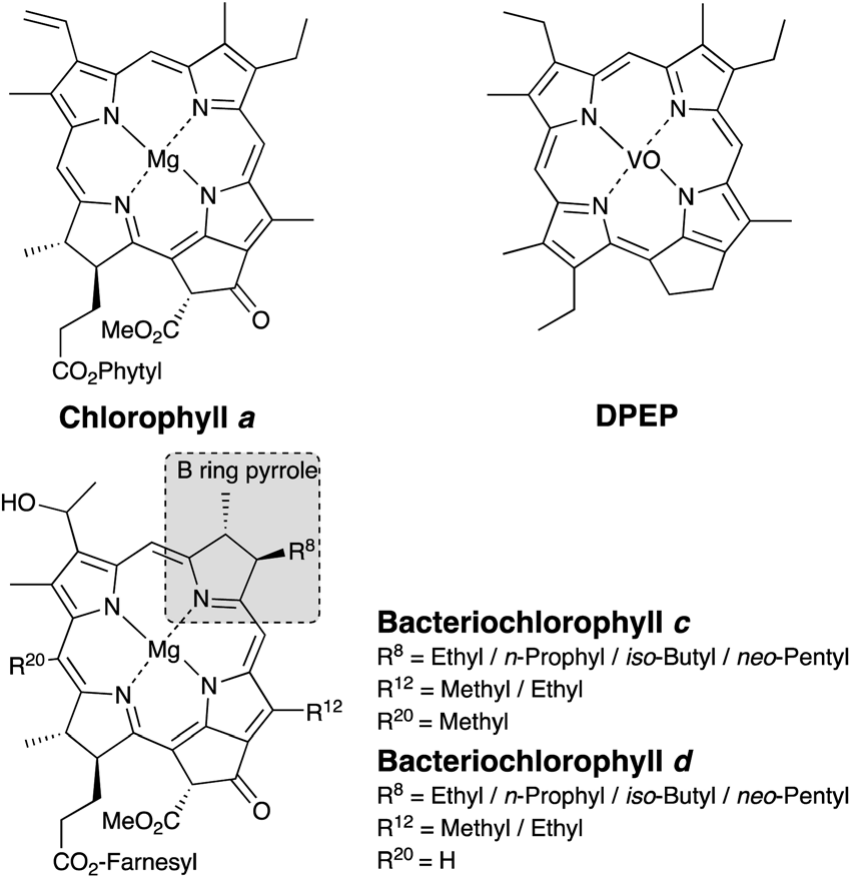
Structures of chlorophyll, bacteriochlorophyll, and deoxophylloerythro-etioporphyrin (DPEP).

Deoxophylloerythroetioporphyrin (DPEP) is one of the typical porphyrins contained in sedimentary rocks, and chlorophyll *a* is thought to be its source material (Chart 1).^1–5^ Since each photoautotroph possesses a different type of chlorophyll,^23^ determination of the source chlorophyll from analysis of the porphyrin in geological samples can lead to an estimation of past photosynthetic organism distributions.^1–21^ The information related to dominant photosynthetic organisms has been previously employed for paleoenvironmental reconstructions, since the paleoenvironment can be estimated from such habitat characteristics.^6–10^

**Fig. 1:**
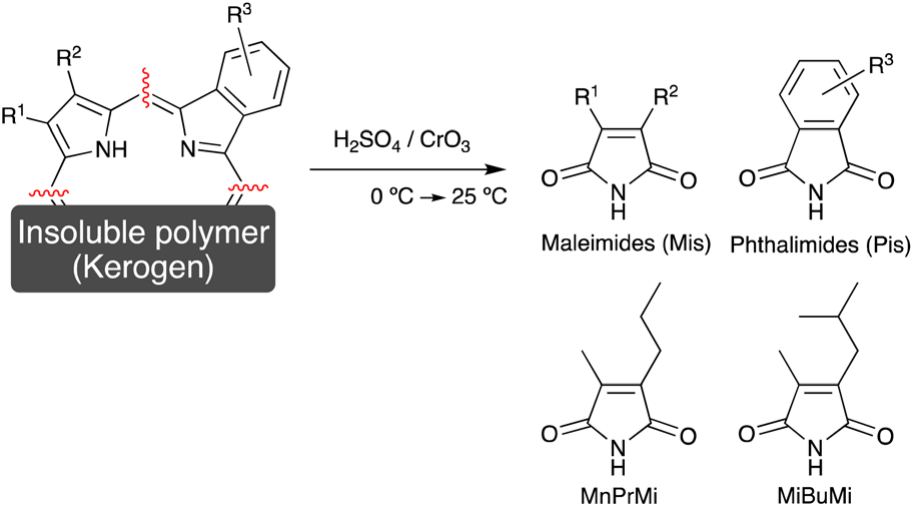
Analytical method for the determination of bonded maleimides and phthalimides in sedimentary rocks by chromic acid oxidation. The structures of 3-methyl-4-propyl- and 3-methyl-4-(2-methylpropyl) maleimide (MnPrMi and MiBuMi) are also shown.

The majority of porphyrins present in sedimentary rocks are trapped within an insoluble and amorphous polymer known as kerogen,^13–22^ and so their extraction with common solvents is challenging. To address this issue, the analysis of maleimides (1*H*-pyrrole-2,5-diones, Mis) derived from the pyrrole skeletons of porphyrins, is promising, since these soluble compounds can be easily extracted and analyzed by gas chromatography-mass spectrometry (GC-MS). The maleimides present in sedimentary rocks can be classified into free or bonded maleimides. The free maleimides are formed during the degradation of chlorophylls in the early diagenesis stage, and are contained in the extracts of sedimentary rocks.^6–10,18^ On the other hand, the bonded maleimides are obtained by the chromic acid oxidation of porphyrins bound to kerogen (Fig. 1).^13–22^ In this chromic acid oxidation method, phthalimides (1,3-dihydro-1,3-dioxoisoindoles, Pis), which are also derived from the porphyrin pyrrole skeletons, are also produced (Fig. 1).

Importantly, the side chain structure of the maleimides and the phthalimides can be used to estimate the source of the fossil porphyrin, *i.e.*, the source chlorophyll.^6–18^ Previous studies have estimated the origin of fossilized chlorophyll based on the analysis of the free maleimides.^9,10^ The 3-methyl-4-propyl-1*H*-pyrrole-2,5-dione (MnPrMi) and 3-methyl-4-(2-methylpropyl)-1*H*-pyrrole-2,5-dione (MiBuMi) detected in extracts of sedimentary rock were considered to be derived from the B-ring pyrrole of bacteriochlorophyll *c*, *d*, or *e* (Chart 1, Fig. 1).^9,10,12^

Bacteriochlorophylls (Chart 1) are chlorophyll-type pigments possessed by anaerobic photosynthetic bacteria, such as green sulfur bacteria.^6–10,23^ Bacteriochlorophylls *c* and *d* are general terms for tetrapyrroles bearing ethyl-, *n*-propyl-, *i*-butyl-, and *neo*-pentyl groups at position 8 on the B-ring pyrrole moiety.^6–10,23^ Previously, MnPrMi and MiBuMi have been detected in samples that contain green sulfur bacterial biomarkers, such as isorenieratane and aryl isoprenoid (a degradation product of isorenieratane).^9,10,12^ Therefore, MnPrMi and MiBuMi, as well as the isorenieratanes, are considered to be evidence of anoxic-sulfidic conditions (at least several mg H_2_S per liter^24^) in the paleo oceanic photic zone.^9,10,12^

However, previous studies into the analysis of maleimides have focused mainly on the free maleimides, with little attention being paid to the bonded maleimides. Our previous studies have elucidated the thermochemical transformation of fossil porphyrins by heating experiments, which are model reaction for thermal maturation. Based on the results, the source chlorophyll of fossil porphyrins was identified, and thermal maturity indices was proposed.^16,17,19–22^ We herein report our examination into the bonded maleimides preserved in kerogen, which can be used as an index of photic zone euxinia in the geologic past. For this purpose, we investigate the optimal conditions for the chromic acid oxidation procedure to extract the bonded maleimides. We also clarify the structural changes of porphyrins associated with experimental thermal pyrolysis, which simulate diagenesis. Finally, we carry out a comparison between the maleimides compositions obtained from the sedimentary rocks and the other proxy compounds of paleo photic zone euxinia, such as the aryl isoprenoids.

Traditionally, extractable indicator molecules such as isorenieratane and aryl isoprenoids have been used to evaluate photic zone euxinia. However, the absolute amounts of these extractable indicator molecules are gradually decreased with thermal maturation because indicator molecules undergo thermal decomposition.^25^ Hence, traditional methods cannot be used to analyze highly matured sedimentary rocks. We propose an index of photic zone euxinia based on the composition of chlorophyll-derived substances incorporated into kerogen. Because kerogen is preserved even in highly maturated sedimentary rocks, our index can therefore be used for highly maturated sedimentary rocks, in which the traditional method could not be used.

## 2 Experimental section

### Geological setting

**Fig. 2:**
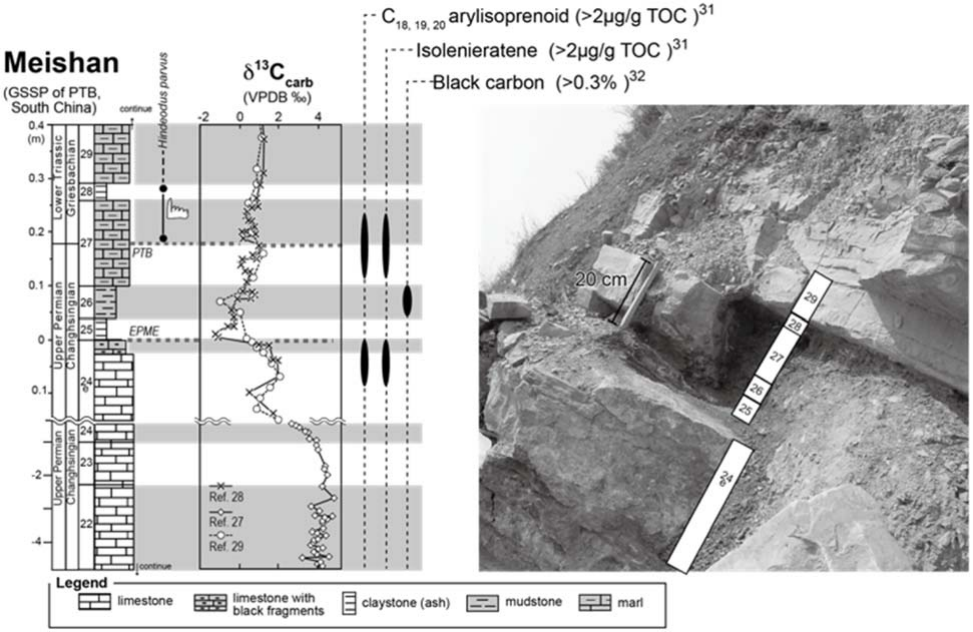
Lithologic column and a photograph of the Meishan section. The stratigraphic positions of the earliest Triassic conodont fossil occurrences (*Hindeodus parvus*),^26^ carbonate carbon isotope (δ^13^C_carb_), stratigraphic positions of high aryl-isoprenoid, isorenieratane, and black carbon^31,32^ are also shown. EPME and PTB mean end-Permian mass extinction event and Permian–Triassic boundary, respectively.

Samples were collected from the Meishan section, which locates in Zhejiang, South China. The Meishan section is one of the best-preserved Permian–Triassic boundary (PTB) sections and is ratified as a GSSP of the PTB interval (Global Boundary Stratotype Section and Point^26^). During the Permian–Triassic period, the Meishan site was in a shallow-water basin of the South China Block, situated in a low latitude region in the eastern paleo-Tethys Ocean. We focused on the 0.6 m thick interval of the Meishan section, which includes the end-Permian-mass extinction (EPME) boundary and the PTB (Fig. 2). This interval is composed of Upper Permian bioclastic limestone (Bed 24e), a white claystone bed (volcanic ash bed; Bed 25), grey-colored claystone (Bed 26), marlstones bearing PTB in its middle part (Bed 27), another claystone (ash) bed (Bed 28), and a marlstone bed (Bed 29). The base of Bed 25 is located at the 0 m point of the measured section shown in Fig. 2. This horizon corresponds to the EPME boundary associated with the sharp negative excursion of carbonate carbon isotope (δ^13^C_carbonate_) (Fig. 2 (ref. 26–29)) and a significant decrease in the number of marine biota fossils.^28,30^

Previous studies have reported significant findings regarding the organic molecules present in the Meishan section. Aryl isoprenoid and isorenieratane, which are known as molecular fossils of the green sulfur bacterial pigment, have been detected in the upper part of Bed 24e and the middle part of Bed 27.^27,29,31^ Increased levels of these organic molecules at horizons indicate an anaerobic sulfidic water environment at the shallow photic zone depth. In addition, the presence of highly concentrated black carbon and polyaromatic hydrocarbons (PAHs) has been reported for Bed 26.^32^ wherein increased levels of these substances could originate from combusted organic matter during frequent wildfires.

### General procedure for analysis of the maleimides and phthalimides present in sedimentary rocks

Powder sample (4.00 g) was added to a mixture of 25% aq. H_2_SO_4_ (5 mL) and 10% aq. CrO_3_ (5 mL) at 0 °C. The resulting mixture was then stirred at 0 °C for 1 h and at 25 °C for 1 h successively. After that, the mixture was extracted with toluene (3 × 10 mL) and the combined organic layer was dried over anhydrous Na_2_SO_4_. The solution was concentrated to a volume of *ca.* 30 μL under a flow of argon gas. The resulting concentrated solution (1 μL) was injected into the GC-MS instrument for analysis.

### Analysis of the maleimides and phthalimides by gas chromatography-mass spectrometry

**Fig. 3:**
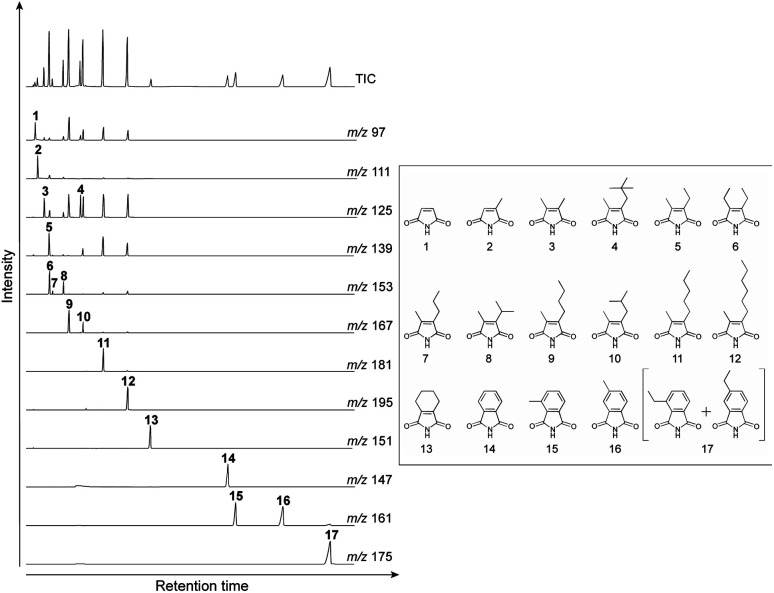
TIC and mass fragmentograms of the authentic samples. These authentic samples have been used in our previous studies.^14–22^ (1) Maleimide (Mi), (2) 2-methyl maleimide (MMMi), (3) 2,3-dimethyl maleimide (DMMi), (4) 2-methyl-3-*neo*-pentyl maleimide (MneoPenMi), (5) 2-ethyl-3-methyl maleimide (EMMi), (6) 2,3-diethyl maleimide (DEMi), (7) 2-methyl-3-*n*-prophyl maleimide (MnPrMi), (8) 2-methyl-3-isoprophyl maleimide (MisoPrMi), (9) 2-*n*-butyl-3-methyl maleimide (MnBuMi), (10) *iso*-butyl-3-methyl maleimide (MisoBuMi), (11) 2-methyl-3-*n*-pentyl maleimide (MnPenMi), (12) 2-methyl-*n*-hexyl maleimide (MnHexMi), (13) tetra-hydrophthalimide (tetraHyPi), (14) phthalimide (Pi), (15) 3-methyl phthalimide (3-MPi), (16) 4-methyl phthalimide (4-MPi), (17) 3- and 4-ethyl phthalimide (3- + 4-EPis).

Gas chromatography-mass spectrometry (GC-MS) was performed on an Agilent 6890-5973 MSD instrument. GC-MS was performed using a DB-FFAP capillary column (60 m × 0.25 mm i.d., 0.25 μm film; Agilent) with helium as the carrier gas at a flow rate of 0.70 mL min^−1^. After holding the oven temperature at 50 °C for 2.0 min, it was increased to 180 °C at a rate of 10 °C min^−1^. Subsequently, the temperature was increased from 180 to 230 °C at a rate of 3 °C min^−1^ and then held at this final temperature for 50 min. Identification and quantification of the maleimides and phthalimides was performed using authentic samples (Fig. 3). The authentic samples of maleimides and phthalimides are not commercially available. In this study, we used the authentic samples which were synthesized by Dr Nomoto and his co-workers. Those authentic samples have been used in his and our previous studies.^14–22^ It should be noted that the individual abundances of the 3- and 4-ethyl phthalimides were not determined under the chromatographic conditions employed herein because they co-eluted during analysis. The peaks corresponding to these compounds were therefore quantified using authentic samples of 3-ethyl phthalimide.

### General procedure for porphyrin pyrolysis

**Chart 2:**
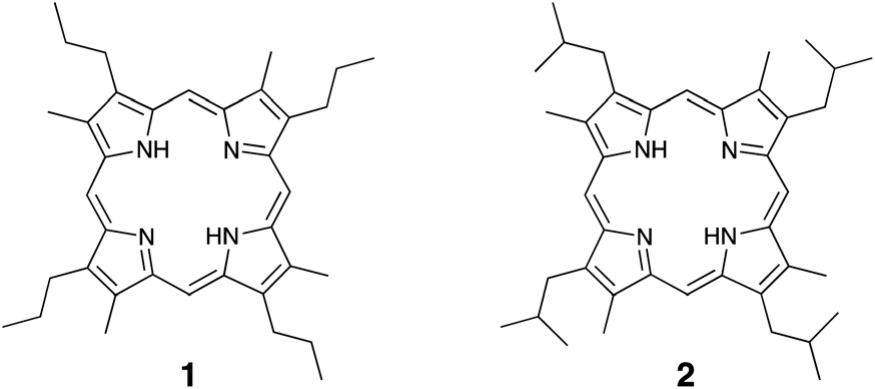
Model porphyrins of the bacteriochlorophylls used for the pyrolysis experiments.

Two model porphyrins, 1 and 2 (Chart 2) were prepared as described in our previous study (detail of the synthetic procedure are described in ESI†).^21,22^ Model porphyrin (*ca.* 0.5 mg) in a degassed sealed glass tube (*ca.* 2 mL capacity) was pyrolyzed at 350 °C for 24 and 72 h. After heating, the sealed glass tube was cooled to room temperature and opened. To each tube containing the pyrolysis products were added 25% aq. H_2_SO_4_ (1 mL) and 10% aq. CrO_3_ (1 mL) at 0 °C, and the reaction was further stirred at 0 °C for 1 h prior to stirring for a further 1 h at 25 °C. After that, the mixture was extracted with toluene (3 × 2 mL) and the combined organic layer was dried over anhydrous Na_2_SO_4_. The solution was concentrated to a volume of *ca.* 100 μL under a flow of argon gas. The resulting concentrated solution (1 μL) was injected into the GC-MS instrument for analysis.

## 3 Results and discussion

**Fig. 4:**
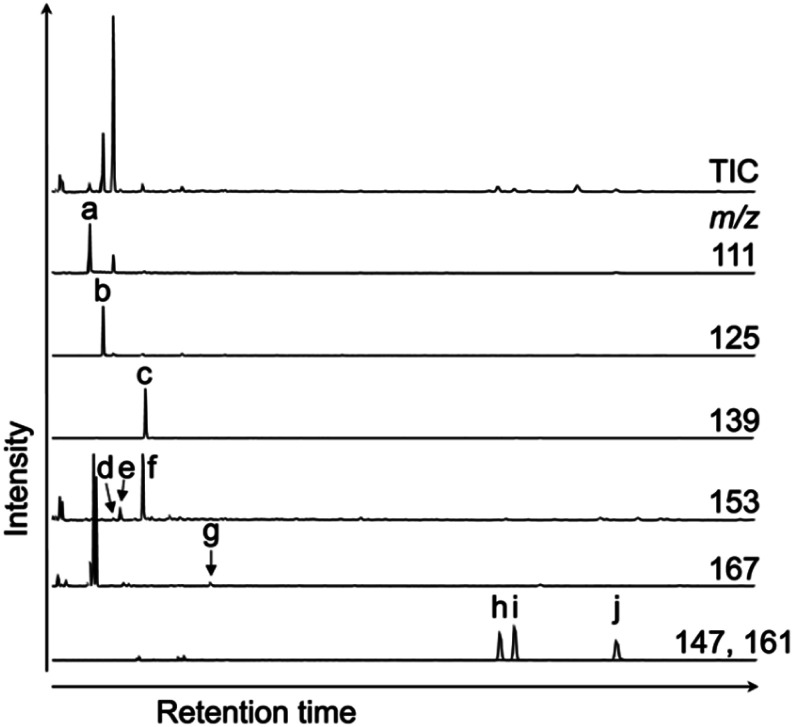
TIC and mass chromatograms of products obtained by the chromic acid oxidation of sedimentary rock (entry 1, Fig. 5 and Table S1,† the sample from the depth −4 to −2 cm of the Meishan section). (a) MMMi, (b) DMMi, (c) EMMi, (d) DEMi, (e) MnPrMi, (f) MiPrMi, (g) MiBuMi, (h) Pi, (i) 3-MPi, and (j) 4-MPi.

**Fig. 5:**
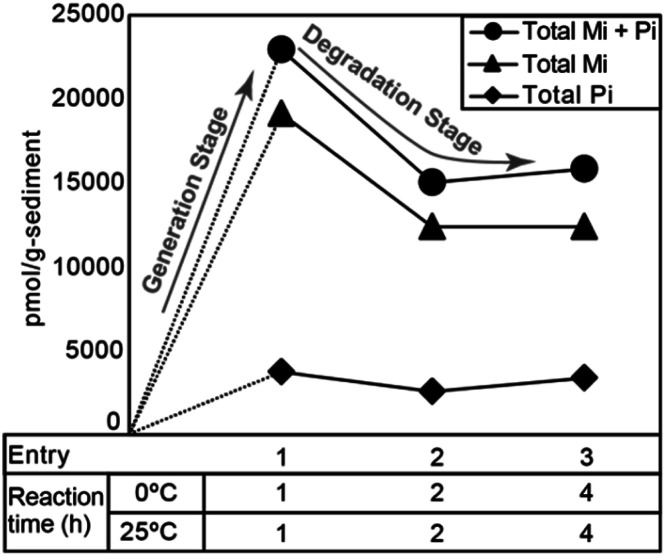
Total yields of maleimide and phthalimide obtained from chromic acid oxidation of sedimentary rock under each reaction condition (Table S1†).

Fig. 4 shows the typical gas chromatograms obtained following the chromic acid oxidation of sedimentary rock (entry 1, Fig. 5, and Table S1,† the sample from the depth −4 to −2 cm of the Meishan section). The top chromatography is a TIC chromatogram of all oxidation products, and the others are mass fragmentgrams showing each maleimide and phthalimide in the chromic acid oxidation products. Identification and quantification of the maleimides and phthalimides were performed using authentic samples (Fig. 3). The peaks of ten maleimides, including MnPrMi and MiBuMi, and three phthalimides were detected (Fig. 4 and Table S1†). Optimization of the chromic acid oxidation conditions was then carried out to maximize the maleimide and phthalimide yields, as outlined in Fig. 5 and Table S1.† The total yields of maleimides and phthalimides were 22 894, 15 039, 15 807 pmol g^−1^-sediment, respectively. It was found that a shorter reaction time (entry 1, 0 °C for 1 h then 25 °C for 1 h) gave a higher yield than those obtained for longer reaction times (*i.e.*, entries 2 and 3, 0 °C for 2 h then 25 °C for 2 h, and 0 °C for 4 h then 25 °C for 4 h). This result suggests that the maleimides and phthalimides formed by the oxidation of kerogen are decreased, since they appear to undergo further oxidative degradation over prolonged reaction times. In entries 2 and 3, there is little difference in the yield of maleimide. This result suggests that chromic acid was almost completely consumed, hence the production and decomposition of maleimide has ended (Fig. 5). In the conditions of longer reaction times (entries 2 and 3), the phthalimide yield did not decrease to the same extent as that of the maleimides (Fig. 5), which implies that the phthalimides exhibit a higher oxidation resistance, likely due to the presence of an aromatic ring in their structure. Further, this result indicates that maleimide and phthalimide formation was complete under the conditions of entry 1, and so these were considered the optimal conditions for obtaining maleimides and phthalimides in high yields.

**Fig. 6:**
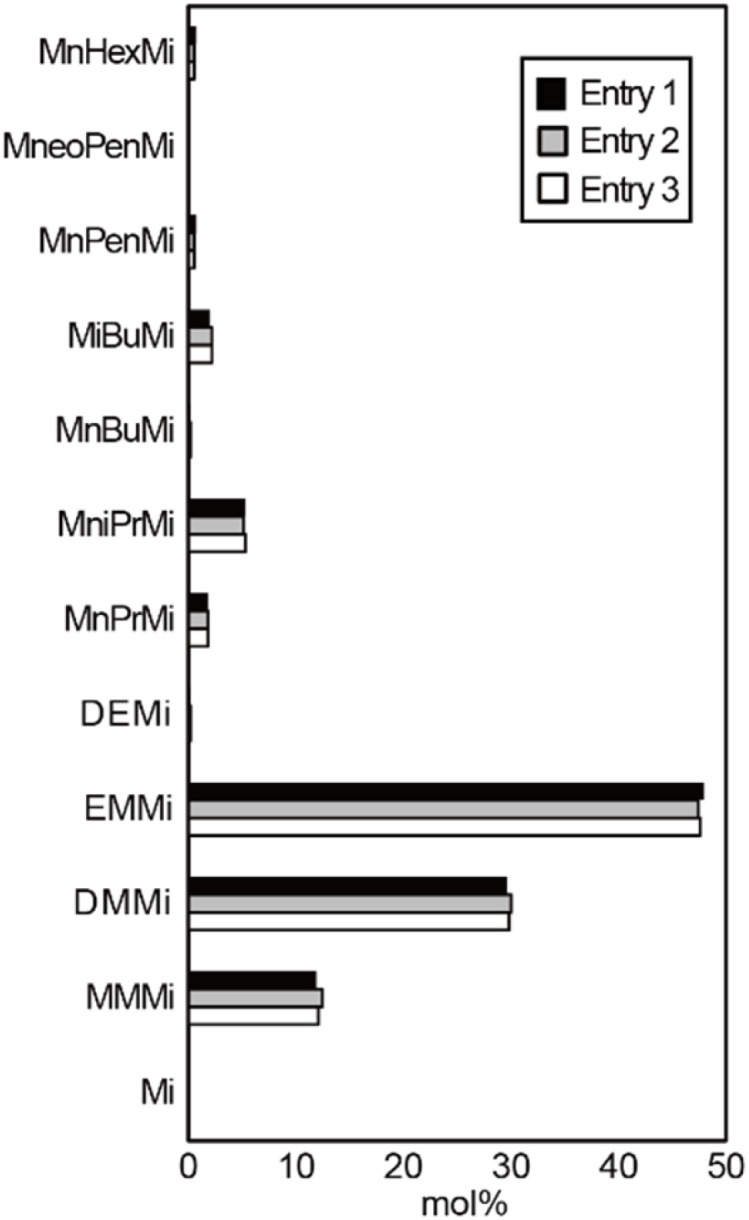
Maleimide contents in the reactions carried out according to entries 1–3 (Table S1†).

Fig. 6 shows the maleimide contents of the reaction mixtures carried out according to entries 1–3 (Table S1†). Despite the large differences in the total yield been these reactions, the overall compositions remained relatively constant, indicating that analysis of the chlorophyll-derived substances based on the chromic acid oxidation method should be based on the product composition rather than the absolute amount of oxidation products. The molecular structure of each maleimide differs only in the alkyl chain. Since the oxidation resistance of the alkyl chains for chromic acid oxidation is expected to be almost the same, it is inferred that the degradations of all maleimides are also the same reaction rate. Therefore, the progress of the oxidation reaction did not affect the composition of the maleimides.

It is known that the side chains of the porphyrins and their related substances present in sedimentary rocks are altered by geothermal pyrolysis.^17,19–21^ Thus, to examine the changes in the side chains of the bacteriochlorophylls, we performed heating experiments using two model porphyrins, 1 and 2 (Chart 2), which were prepared as described in our previous study.^21,22^ We designed these model porphyrins based on simple structures to clarify the changes taking place in the pyrrole B-ring pyrroles of the bacteriochlorophylls. Since the pyrolysis products of porphyrins are insoluble polymeric materials, the pyrolysis products were converted into maleimides and phthalimides by the chromic acid oxidation. After heating at 350 °C for 3 days, MnPrMi and MiBuMi, which are the maleimides corresponding to model porphyrins 1 and 2, were detected in mole fractions of 50 and 64 mol%, respectively (Table 1). These results indicate that the pyrroles derived from bacteriochlorophylls are altered by thermal maturation. In addition, MMMi and DMMi were the major products obtained from the pyrolysis of model porphyrins 1 and 2. It was therefore considered that DMMi was predominantly produced due to cleavage at the aryl position of the alkyl chain, where relatively stable radicals are easily generated. Similar dealkylation reactions have been observed in the pyrolysis of etioporphyrin, which contains the 3-ethyl-4-methyl-pyrrole.^19^ EMMi, which is derived from 3-ethyl-4-methyl-pyrrole and is found in multiple types of chlorophylls, was also produced (≤3 mol%) during the pyrolysis of porphyrins 1 and 2.

**Table 1:** Mole fractions of the maleimides and phthalimides present following the pyrolysis of porphyrins 1 and 2 at 350 °C

Starting material	Mole fractions of the Mis and Pis (mol%)			
	Porphyrin 1		Porphyrin 2	
Heating time (days)	1	3	1	3
MMMi	20	20	6	15
DMMi	20	20	6	11
EMMi	3	3	<1	1
MnPrMi	49	50	<1	2
MiBuMi	<1	<1	84	64
Other maleimides	1	1	<1	<1
Phthalimides	6	6	3	8

We therefore proposed the Maleimide Index (MI) to evaluate the increases in anaerobic photoautotrophs in the anoxic-sulfidic photic zone conditions of the paleo ocean as follows:
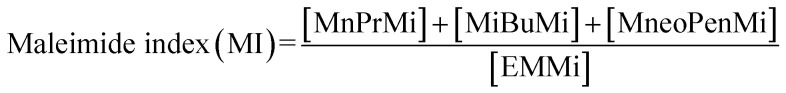


The MI represents the ratio of pyrroles derived from the B-ring pyrroles of bacteriochlorophylls to 3-ethyl-4-methyl-pyrrole which is present in various chlorophylls, including bacteriochlorophylls and chlorophyll *a*. Thus, MI approximates the ratio of green sulfur bacteria to total photosynthetic organisms. The pyrolysis experiments of model porphyrins 1 and 2 suggested that EMMi was formed from the thermal maturation of bacteriochlorophylls (Table 1). However, the mole fraction of the produced EMMi was low (<3%). Since EMMi is present in larger amounts in sedimentary rocks than MnPrMi, MiBuMi, and MneoPenMi, it would be expected that even if the EMMi content is increased during pyrolysis, this indicator would have little effect on the overall MI.

**Fig. 7:**
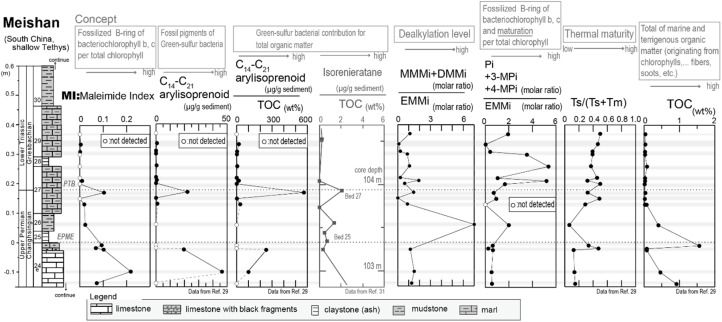
Analytical results of sedimentary rocks, which were collected from the Meishan section in China.

**Fig. 8:**
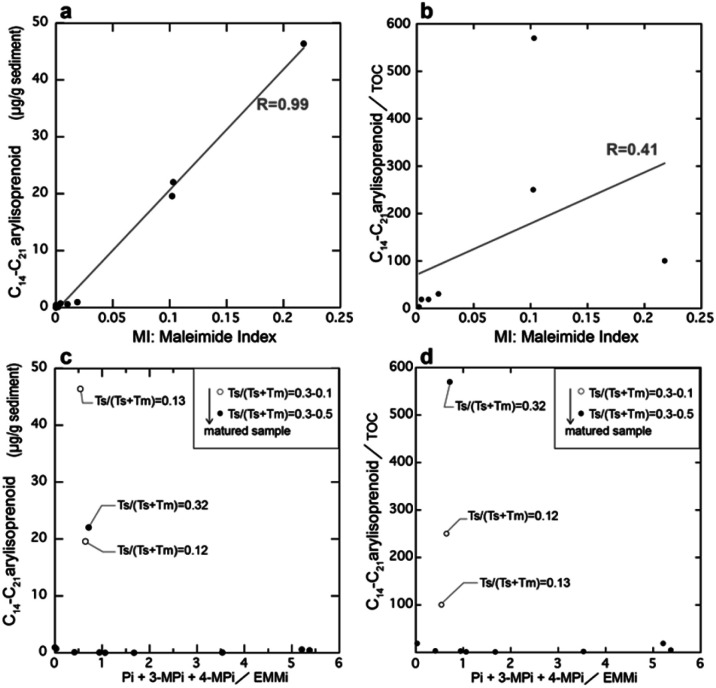
Correlation between the composition ratios of the maleimides and the concentrations of the arylisoprenoids. (a) The maleimide index (MI) and the concentrations of the arylisoprenoids, (b) the MI and the arylisoprenoid/TOC content, (c) the ratio of phthalimides (Pi + 3-MPi + 4-MPi) to EMMi and the concentration of arylisoprenoids, and (d) the ratio of phthalimides (Pi + 3-MPi + 4-MPi) to EMMi and the arylisoprenoid/TOC content.

Next, we examined the correlation between MI and the other indicators of anaerobic green sulfur bacteria, namely isorenieratane and the aryl isoprenoids. Fig. 7 shows our analytical results and previously reported data^29,31^ obtained from the Meishan section in South China. The Meishan section is a shallow marine Permian–Triassic boundary that records the severe mass extinction event and photic zone euxinia through increases in the isorenieratane and aryl isoprenoid contents.^27,31,32^ It should be noted here that the samples analyzed herein were the same as those employed to measure the aryl isoprenoid contents in our previous study.^29^ As shown in Fig. 7, the MI values increased in the lower and middle parts of the studied interval (upper part of Bed 24e and middle part of Bed 27). These horizons also show increases in the aryl isoprenoid content and the aryl isoprenoids per total organic carbon (TOC) ratio.^29,31^ Indeed, high values of isorenieratane and isorenieratane/TOC have been previously detected in the same horizons,^29,31,32^ as outlined in Fig. 2 and 7. These results suggest that the newly proposed MI for use in sedimentary rocks represents the increase in anaerobic photoautotrophs (such as green sulfur bacteria) during the depositional time. The synchronous variations were also confirmed in the cross-plots between the MI and the aryl isoprenoid content using the same samples, and strong positive correlations were observed (*R* > 0.9, Fig. 8a). In contrast, the correlation between the MI and the aryl isoprenoids/TOC ratio was low (*R* = 0.4, Fig. 8b), which may be attributed to the compositional differences of EMMi and the TOC in terms of the data normalization. The TOC value reflects the amount of organic matter in the sedimentary rock which is originated not only from chlorophylls but also from various organic molecules such as lipids, proteins, amino acids *etc.* In this study, Bed 26 contains black carbon, such as soot and charcoals. Therefore, organic matter originated from photosynthetic organisms in Bed 26 is diluted by black carbon. Indeed, the variation in TOC value is consistent with the variation in black carbon content in previous studies^32^ but is inconsistent with the variation in MI and aryl isoprenoid values (Fig. 7). This result indicates that TOC values do not necessarily reflect the amount of photosynthetic organisms. On the other hand, the MI method is normalized by EMMi derived from pyrrole, which is possessed by all chlorophylls. Therefore, the MI method is not affected by organic matter other than photosynthetic organisms. Furthermore, the aryl isoprenoids could be derived from additional carotenoid sources in addition to green sulfur bacteria, thereby causing discrepancies in the variations between the MI and the aryl isoprenoid content. We therefore considered that use of the MI is one of the most accurate means to estimate actual photoautotroph composition in the paleo photic zone.

Based on the thermal maturity index of Ts/(Ts + Tm),^33^ (Ts and Tm are C_27_ 18α(*H*)-22,29,30-trisnorneohopane and C_27_ 17α(*H*)-22,29,30-trisnorhopane, respectively), the maturity level of the Meishan section was found to be low in the lower limestone beds (Ts/(Ts + Tm) < 0.2) and high in the middle to upper muddy strata (Ts/(Ts + Tm) = 0.2–0.5), with the exception of Bed 26 (Fig. 7). This can be accounted for by considering that Bed 26 contains black carbon and polyaromatic hydrocarbons (PAHs) attributed to wildfires,^31^ thereby resulting in a significantly altered organic matter content. In addition, as shown in Fig. 7, Bed 26 contained significantly higher MMMi and DMMi contents compared to EMMi, and this result is consistent with the results of our experiments, wherein dealkylation took place during sample pyrolysis.

Our previous study reported that aromatization of the 3-methyl-4-*n*-propyl-pyrrole or the 3-*i*-butyl-4-methyl-pyrrole may result in the formation of a benzopyrrole.^22^ Thus, we also examined the correlation between the ratio of EMMi to the benzopyrrole-derived phthalimides,^34^ and the vertical plots of isorenieratane and aryl isoprenoids (Fig. 7). However, the ratio of phthalimides to EMMi did not increase with the aryl isoprenoids (Fig. 7, 8c and d). The ratio of phthalimides to EMMi^34^ gave higher values in the upper part of the studied interval, which is characterized by a high maturity level (as derived from Ts/(Ts + Tm)). Furthermore, for Bed 26, which has a high contribution from MMMi and DMMi, this horizon exhibits a slightly higher phthalimide to EMMi ratio. These high values suggest a large contribution from the benzopyrrole unit produced by thermal alteration of chlorophylls other than bacteriochlorophylls. Possible reasons for these high values are mainly two. First, some organic matter sources with high benzopyrrole unit composition are derived in Bed 26 coinciding with combustion origin organic matters. Another reason is that benzopyrrole unit formation in the sediments proceeded during the thermal maturation and result in high values in the upper part interval of the Meishan section. The formation of the benzopyrrole unit during thermal treatment could likely be attributed to the Diels–Alder reaction of the 3-methyl-4-vinylpyrrole unit,^19^ or aromatization *via* the *trans* alkylation and cyclization of 3-ethyl-4-methylpyrrole.^20^ Overall, these multiple controls indicate that the phthalimide/EMMi ratio is not suitable for evaluating the contribution of green sulfur bacteria to the total photosynthetic biomass.

## 4. Conclusions

We proposed the MI to explore the contribution of anaerobic photosynthetic bacteria in the anoxic ocean photic zone using maleimides derived from bacteriochlorophylls. Our index (MI), which uses the bonded maleimides, can be analyzed without additional pretreatment other than chromic acid oxidation. This methodology is significantly more straightforward than that based on the analysis of free maleimides and fossil porphyrins, which requires the extraction of sedimentary rocks through column chromatographic fractionation. Traditional methods, such as using green sulfur bacterial pigment molecular fossils as indicator molecules are normalized by TOC. The TOC value reflects the amount of organic matter in the sedimentary rock which is originated not only from chlorophylls but also from various organic molecules such as lipids, proteins, amino acids *etc.* TOC values do not necessarily reflect the variability of photosynthetic organisms. In fact, Bed 26, which contains combustion-derived black carbon, may have affected the TOC value. On the other hand, our MI method is normalized by EMMi which is contained in all chlorophyll. Thus, compared with traditional methods, our MI method enables us to determine the actual photosynthetic composition using only chlorophyll-derived compounds. This method should therefore contribute to elucidation of the paleo ocean ecosystem and the oceanic structures present at the ocean anoxia in the geologic past.

## Conflicts of interest

There are no conflicts to declare.

## Supplementary Material

RA-012-D2RA04702K-s001
