# Supplementary material for: Maleimide index: a paleo-redox index based on fragmented fossil-chlorophylls obtained by chromic acid oxidation
Source: RSC Adv. 2022 Oct 31;12(48):31061–7. doi: 10.1039/d2ra04702k (PMC9620498; doi:10.1039/d2ra04702k)
Supplement: RA-012-D2RA04702K-s001 [file RA-012-D2RA04702K-s001.pdf]

Electronic Supplementary Material (ESI) for RSC Advances.
This journal is © The Royal Society of Chemistry 2022

Electronic Supplementary Information

Maleimide Index: A paleo-redox index based on the fragmented fossil-chlorophylls obtained by chromic acid oxidation

Kenta Asahina[a*], Satoshi Takahashi[b], Ryosuke Saito[c,d], Kunio Kaiho[e] and Yasuhiro Oba[f]

[a]Research Institute of Geo-Resources and Environment, Geological Survey of Japan, National Institute of Advanced Industrial Science and Technology, Central 7, 1-1-1 Higashi, Tsukuba, Ibaraki 305-8567, Japan

E-mail: kenta-asahina@aist.go.jp

[b]Department of Earth and Environmental Sciences, Nagoya University, Furo-cho, Chikusa-ku, Nagoya, Aichi 464-8601, Japan

[c]Department of Geosphere Sciences, Yamaguchi University, 1677-1 Yoshida, Yamaguchi City, Yamaguchi 753-8512, Japan

[d]Japan Science and Technology Agency, PRESTO, 4-1-8 Honcho, Kawaguchi, Saitama 332-0012, Japan

[e]Department of Earth Science, Tohoku University, Aoba-aza, Aramaki, Aoba-ku, Sendai, 980-8578, Japan.

[f]Institute of Low Temperature Science, Hokkaido University, N19W8, Kita-ku, Sapporo, Hokkaido 060-0819, Japan

Table S1. Yields of the maleimides and phthalimides obtained under different chromic acid oxidation conditions (the sample from the depth -4 to -2cm of the Meishan section).

		Entry 1		Entry 2		Entry 3	
Reaction time (h)	0 ºC	1		2		4	
	25 ºC	1		2		4	
		Yield (pmol/g-sediment)	Composition (%)	Yield (pmol/g-sediment)	Composition (%)	Yield (pmol/g-sediment)	Composition (%)
Maleimide	Mi	ND	ND	ND	ND	ND	ND
	MMMi	2258	9.9	1538	10.2	1489	9.4
	DMMi	5650	24.7	3723	24.8	3700	23.4
	EMMi	9161	40.0	5879	39.1	5883	37.2
	DEMi	2	Tr	1	Tr	1	Tr
	MnPrMi	359	1.6	225	1.5	228	1.4
	MiPrMi	1013	4.4	643	4.3	655	4.1
	MnBuMi	10	Tr	5	Tr	5	Tr
	MiBuMi	382	1.7	264	1.8	279	1.8
	MnPenMi	120	0.5	64	0.4	60	0.4
	M*neo*PenMi	ND	ND	ND	ND	ND	ND
	MnHexMi	141	0.6	67	0.4	75	0.5
	tetraHyPi	ND	ND	ND	ND	ND	ND
Phthalimide	Pi	1926	8.4	1368	9.1	1757	11.1
	3-MPi	835	3.6	569	3.8	748	4.7
	4-MPi	733	3.2	486	3.2	670	4.2
	3- + 4-EPi	295	1.3	208	1.4	257	1.6
Total		22894	100.0	15039	100.0	15807	100.0

Tr = Trace amount (<0.01%)

ND = not detected

Table S2. Sedimentary geochemical data from the Meishan section in China

	Sample No.	1	2	3	4	5	6	7	8	9	10	11	12	13	14	15
	Depth (cm)	37–39	33.5–36.5	31–33	30–33.5	26–29	22–24	21–23	19–20	17–19	15–16	13–15	6–7	−2.5	−10	−14–−12
Composition (%)	Mi	ND	ND	ND	ND	ND	ND	ND	8.71	0.88	ND	ND	ND	0.58	3.54	ND
	MMMi	14.29	2.50	3.47	2.22	3.42	4.61	5.89	6.35	9.39	ND	15.29	11.30	16.35	13.74	11.14
	DMMi	6.32	5.46	9.82	13.57	10.72	5.23	14.23	6.25	33.57	ND	31.29	58.49	22.57	29.81	20.77
	EMMi	18.49	88.09	57.12	18.04	13.26	41.70	10.46	20.15	29.67	43.89	52.43	9.98	33.96	28.91	25.21
	DEMi	ND	ND	ND	ND	ND	ND	ND	ND	0.06	0.32	ND	ND	0.01	0.01	0.01
	MnPrMi	ND	0.35	ND	ND	ND	ND	ND	ND	ND	ND	ND	ND	1.82	1.27	0.69
	MiPrMi	ND	0.73	ND	ND	ND	ND	ND	15.46	ND	ND	ND	ND	0.25	0.05	25.21
	MnBuMi	ND	ND	ND	ND	ND	ND	ND	ND	0.35	ND	ND	ND	0.03	0.01	ND
	MiBuMi	ND	ND	ND	ND	ND	ND	ND	ND	3.06	ND	1.00	0.23	1.66	5.03	1.14
	MnPenMi	ND	0.16	ND	ND	ND	ND	ND	ND	ND	ND	ND	ND	ND	ND	ND
	MneoPenMi	ND	0.02	0.11	ND	ND	ND	0.11	ND	ND	ND	ND	ND	ND	ND	ND
	MHexMi	ND	ND	ND	ND	ND	ND	ND	ND	ND	ND	ND	ND	0.61	0.51	ND
	tetraHyPi	1.71	ND	5.93	ND	ND	2.67	14.84	9.32	ND	14.43	ND	ND	0.07	0.06	ND
	Pi	13.38	1.05	9.03	22.58	33.58	13.89	27.09	16.10	10.88	35.36	ND	3.97	12.01	9.06	8.11
	3-MPi	8.29	0.65	5.64	15.42	14.37	12.40	11.03	6.73	5.05	ND	ND	10.09	4.99	3.28	3.07
	4-MPi	14.19	0.99	8.87	25.72	23.26	17.88	16.35	10.93	5.32	6.00	ND	5.93	4.93	3.27	3.24
	3-+4-EPi	23.33	ND	ND	2.46	1.39	1.63	0.00	ND	1.78	0.00	ND	ND	0.16	1.46	1.41
Maleimide Index (MI)[a]		ND	0.004	0.002	ND	ND	ND	0.010	ND	0.103	ND	0.019	0.023	0.102	0.218	0.073
$\frac{[Pi]+[3-Pi]+[4-Pi]}{[EMMi]}$		1.940	0.031	0.412	3.533	5.371	1.059	5.209	1.675	0.716	0.942	ND	2.005	0.646	0.540	0.572
$C_{14\text{-}19}$ arylisoprenoid [b)] (μg/g sediment)		ND	0.719	0.101	0.060	0.409	0.035	0.561	0.018	22.05	0.063	0.971	ND	19.59	46.39	ND
$\frac{[C_{14-19}\text{ arylisoprenoid}]}{TOC}$ [b)]		ND	18.91	3.219	1.930	4.859	1.198	18.99	0.910	569.8	2.774	30.57	ND	250.1	100.4	ND
$\frac{[MMMi]+[DMMi]}{[EMMi]}$		1.114	0.090	0.233	0.875	1.067	0.236	1.924	0.625	1.448	ND	0.888	6.996	1.146	1.506	1.266
$\frac{[Ts]}{[Ts]+[Tm]}$ [b)]		0.500	0.468	0.392	0.393	0.371	0.456	0.323	0.502	0.323	0.489	0.289	0.063	0.126	0.136	0.147
TOC (wt%) [b)]		0.039	0.038	0.031	0.031	0.084	0.030	0.030	0.020	0.039	0.023	0.072	0.405	0.078	0.462	0.918

a) Maleimide Index = [MnPrMi + MiBuMi + MneoPenMi]/EMMi

b) Data from reference S4

ND = not detected

Instrumentation

Thin-layer chromatography (TLC) was carried out using Merck pre-coated plates (silica gel $60F_{254}$, Art 57715, 0.25 mm). Flash column chromatography was performed using a Biotage Isolera® equipped with Biotage Sfär Silica HC D columns or SNAP Ultra Silica columns and hexane/ethyl acetate as the eluent. For flash column chromatography of porphyrins, silica gel 60N (spherical, neutral, 63-210 μm) from Kanto Chemical Co., Ltd. was used. Silica gel preparative TLC (PTLC) was performed on Merck silica gel $60PF_{254}$ (Art 7749). 1H NMR and 13C NMR were measured on a Bruker Avance III 500 spectrometer. High resolution mass spectroscopy was carried out using a Thermo Scientific Exactive spectrometer (electrospray ionization, ESI) and a JEOL JMS-700 spectrometer (electron ionization, EI). Gas chromatography–mass spectrometry (GC-MS) was performed on an Agilent 6890-5973 MSD instrument. Before the GC-MS measurement, auto-tuning was performed to ensure that the measurement system was working properly. Before measuring the samples, a mixture of authentic samples for maleimides and phthalimides was measured to confirm the detection sensitivity of each compound. Melting point (Mp) determinations were performed by using a Yanako MP-S3 instrument and are uncorrected.

Preparation of the Pyrroles

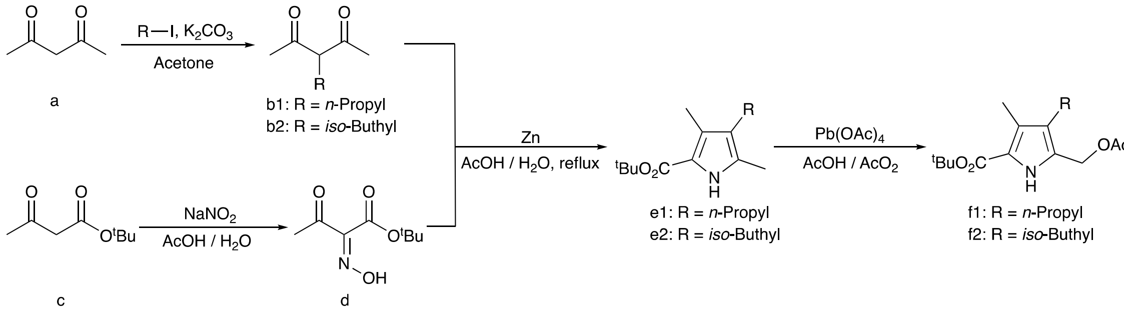

Scheme S1. Synthesis of the pyrroles.

*Synthesis of 3-n-propyl-2,4-pentanedione **b1***

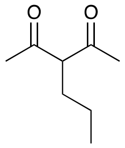

A mixture of 1-iodopropane (14.31 g, 84.18 mmol), 2,4-pentanedione (7.00 g, 69.92 mmol), and K_2CO_3 (9.71 g, 70.26 mmol) in acetone (14 mL) was stirred overnight at reflux. After that, the reaction mixture was added to water (100 mL), and the products were extracted with diethyl ether (×3). The combined organic layer was washed water (×3), an aqueous saturated citric acid solution (×3), and brine (×1), then dried over anhydrous Na_2SO_4. After filtration, the solvent was removed in vacuo, and

the residue was purified by vacuum distillation to give the keto-enol tautomer of 3-*n*-propyl-2,4-pentanedione **b1** (5.07 g, 51.0%) as a light-yellow oil. The obtained product was used in the next step without further purification. δH($CDCl_3$) 0.94 (6H, m), 1.28 (2H, m), 1.43 (2H, m), 1.83 (2H, m), 2.13 (6H, s), 2.17 (6H, s) and 3.63 (1H, t, J = 15 Hz); δC($CDCl_3$) 13.9, 14.0, 20.8, 22.9, 23.8, 29.0, 29.6, 30.4, 68.9, 110.5, 191.1 and 204.6; HRMS (ESI) *m/z* 165.0885 (165.0886 calcd for $C_8H_{14}O_2Na$ $[M+Na]^+$).

Synthesis of 3-iso-butyl-2,4-pentanedione ***b2***

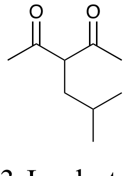

3-Iso-butyl-2,4-pentanedione **b2** was synthesized from a mixture of 1-iodo-2-methylpropane (30.94 g, 168.13 mmol), 2,4-pentanedione (14.00 g, 139.83 mmol), K_2CO_3 (19.32 g, 139.79 mmol), and acetone according to the procedure described for compound **b1**. Isolation of the crude product delivered 7.97 g (36.5%) of the keto-enol tautomer of **b2** as a light-yellow oil. δH($CDCl_3$) 0.91 (6H, d, J = 5.0 Hz), 0.98 (1H, d, J = 10 Hz), 1.48 (1H, m), 1.74 (2H, t, J = 10 Hz), 2.18 (6H, s), 2.29 (0.5H, s) and 3.73 (1H, t, J = 15 Hz); δC($CDCl_3$) 19.1, 19.7, 22.2, 22.3, 23.3, 26.4, 27.8, 28.9,29.9, 31.9, 36.9, 67.2, 74.4, 99.6, 172.2, 191.5, 196.9 and 204.4; HRMS (ESI) *m/z* 179.1041 (179.1043 calcd for $C_9H_{16}O_2Na$ $[M+Na]^+$).

Synthesis of 1,1-dimethylethyl-2-(hydroxyimino)-3-oxobutanoate ***d***

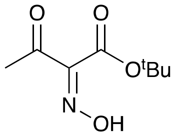

An aqueous solution (51.3 mL) of $NaNO_2$ (18.33 g, 265.6 mmol) was added dropwise to an acetic acid solution (51.3 mL) of *tert*-butyl acetoacetate **c** (30.01 g, 189.7 mmol) at 10 ºC. Subsequently, the reaction mixture was allowed to warm to room temperature, and after stirring for 1 h at this temperature, water (150 mL) was added. The products were then extracted with dichloromethane (×3) and the combined organic layer was washed with water (×2), 5% aq. $NaHCO_3$ (×3), and brine (×1). The organic solution was dried over anhydrous Na_2SO_4, filtered, and the solvent was removed in vacuo. The obtained residue was purified using the Isolera® (SNAP Ultra, hexane/ethyl acetate = 19:1 → 3:2) to give the desired 1,1-dimethylethyl-2-(hydroxymino)-3-oxobutanoate **d** (31.70 g, 89.3%) as a colorless oil. δH($CDCl_3$) 1.56 (9H, s), 2.38 (3H, s) and 10.11 (1H, br s); δC($CDCl_3$) 25.3, 28.1, 85.1, 151.5, 161.4 and 194.5; HRMS (ESI) *m/z* 210.0734 (210.0737 calcd for $C_8H_{13}O_4Na$ $[M+Na]^+$).

Synthesis of pyrrole ***e1***

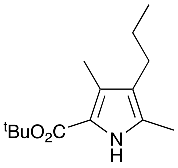

An aqueous acetic acid solution (AcOH 16.2 mL, H_2O 10.8 mL) of 1,1-dimethylethyl-2-(hydroxymino)-3-oxobutanoate **d** (5.98 g, 31.94 mmol) and zinc powder (4.39 g, 67.15 mmol) was added dropwise to an acetic acid solution of 3-*n*-propyl-2,4-pentanedione **b1** (5.00 g, 35.16 mmol) while maintaining the temperature at 70 ºC. The reaction mixture was then heated under reflux for 1 h, and the hot solution was poured into ice water (700 mL). The resulting solid was collected by filtration then dissolved in ethyl acetate. After washing the organic solution was washed with water (×2), saturated $NaHCO_3$ aq. (×3), and brine (×1), it dried over anhydrous Na_2SO_4, filtered, and the solvent was removed in vacuo. The obtained residue was purified using the Isolera® (SNAP Ultra, hexane/ethyl acetate = 49:1 → 4:1) to give the desired pyrrole **e1** (1.54 g, 18.5%) as a light-yellow solid. Mp 111.0-112.0ºC; δH($CDCl_3$) 0.90 (3H, t, *J* = 15 Hz), 1.44 (2H, m), 1.56 (9H, s), 2.18 (3H, s), 2.23 (3H, s), 2.32 (2H, t, *J* = 15 Hz) and 8.94 (1H, br s); δC($CDCl_3$) 10.6, 11.5, 13.9, 24.0, 26.1, 28.6, 80.0, 118.0, 122.0, 126.3, 128.8 and 161.3; HRMS (ESI) *m/z* 260.1617 (260.1621 calcd for $C_{14}H_{23}NO_2Na$ $[M+Na]^+$).

Synthesis of pyrrole ***e2***

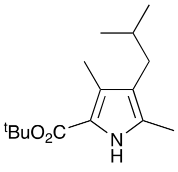

Pyrrole **e2** was synthesized from 1,1-dimethylethyl-2-(hydroxymino)-3-oxobutanoate **d** (5.98 g, 31.94 mmol), zinc powder (4.39 g, 67.15 mmol), and 3-iso-butyl-2,4-pentanedione **b2** (5.49 g, 35.14 mmol) according to the procedure described for compound **e1**. Isolation of the crude product delivered 1.29 g (16.1%) as a yellow solid. Mp 132.0-133.5ºC; δH($CDCl_3$) 0.87 (3H, s), 0.89 (3H, s), 1.56 (9H, s), 1.70 (1H, m), 2.17 (3H, s), 2.21 (2H, d, *J* = 5.0 Hz), 2.22 (3H, s) and 8.66 (1H, br s); δC($CDCl_3$) 10.9, 11.8, 22.5, 28.6, 30.0, 33.4, 80.0, 118.0, 121.3, 126,6, 129.2 and 161.4; HRMS (ESI) *m/z* 274.1773 (274.1778 calcd for $C_{15}H_{25}NO_2Na$ $[M+Na]^+$).

*Synthesis of pyrrole **f1***

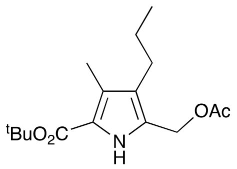

$Pb(OAc)_4$ (1.98 g, 4.47 mmol) was added to an acetic acid/acetic anhydride solution (AcOH 12.8 mL, Ac_2O 0.64 mL) of pyrrole **e1** (1.00 g, 4.21 mmol) under argon gas. After stirring for 1 h at room temperature, the reaction mixture was diluted with dichloromethane (30 mL), and washed with water (×3), 5% aq. $NaHCO_3$ (×3), and brine (×1). The organic layer was dried over anhydrous Na_2SO_4, filtered, and the solvent was removed in vacuo. The obtained residue was purified using the Isolera® (Sfär Silica HC D, hexane/ethyl acetate = 19:1 → 7:3) to give the desired pyrrole **f1** (0.58 g, 46.6%) as light-yellow solid. Mp 97.0-98.0ºC; δH($CDCl_3$) 0.92 (3H, t, J = 15 Hz), 1.46 (2H, m), 1.56 (9H, s), 2.06 (3H, s), 2.24 (3H, s), 2.41 (2H, t, J = 15 Hz), 5.01 (2H, s) and 8.94 (1H, br s); δC($CDCl_3$) 10.4, 13.9, 21.0, 24.4, 25.9, 28.5, 57.1, 80.6, 120.5, 125.0, 125.4, 126.3, 161.0 and 171.6; HRMS (ESI) *m/z* 318.1673 (318.1676 calcd for $C_{16}H_{25}NO_4Na$ $[M+Na]^+$).

*Synthesis of pyrrole **f2***

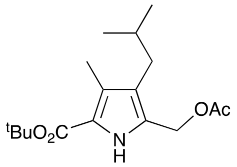

Pyrrole **f2** was synthesized from pyrrole **e2** (0.52 g, 2.07 mmol) and $Pb(OAc)_4$ (0.972 g, 2.19 mmol) according to the procedure described for compound **f1**. Isolation of the crude product delivered 0.330 g (51.7%) as a yellow solid. Mp 100.5-102.0ºC; δH($CDCl_3$) 0.88 (3H, s), 0.90 (3H, s), 1.56 (9H, s), 1.71 (1H, m), 2.06 (3H, s), 2.23 (3H, s), 2.29 (2H, d, J = 10 Hz), 5.00 (2H, s) and 8.97 (1H, br s); δC($CDCl_3$) 10.7, 20.9, 22.4, 28.5, 30.0, 33.1, 57.2, 80.6, 120.5, 124.1, 125.7, 126.7, 161.0 and 171.6; HRMS (ESI) *m/z* 332.1827 (332.1832 calcd for $C_{17}H_{27}NO_4Na$ $[M+Na]^+$).

Synthesis of the Porphyrins

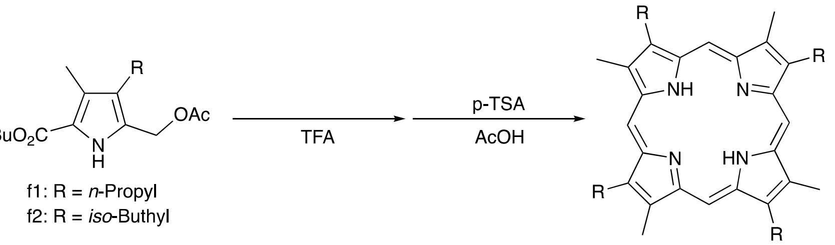

Scheme S2. Synthesis of porphyrins **1** and **2**.

*Synthesis of porphyrin **1***

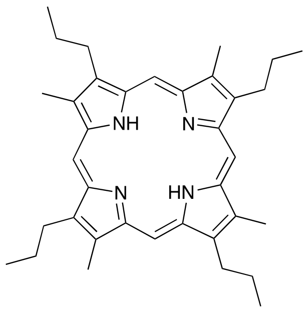

A TFA solution (5.3 mL) of pyrrole **f1** (300 mg, 1.02 mmol) was stirred for 10 min at room temperature under Ar in the dark, after which time the solvent was removed in vacuo. The residue was then dissolved in AcOH (4.0 mL), and *p*-toluenesulfonic acid monohydrate (19.5 mg, 0.103 mmol) was added under Ar in the dark. After stirring the resulting solution for 12 h under Ar, it was stirred for a further 12 h under air. Subsequently, the reaction mixture was diluted with $CHCl_3$ (30 mL), then washed with water (×3), saturated $NaHCO_3$ aq. (×3), and brine (×1). After drying the organic layer over anhydrous Na_2SO_4, it was filtered, and the solvent was removed in vacuo. The residue was purified by column chromatography (activated alumina 75 μm, Fujifilm Wako Pure Chemical Co., Ltd.), $CHCl_3$) and the red band was collected. Finally, the dark red product was subjected to flash column chromatography (silica gel, hexane/ethyl acetate = 19:1) to obtain porphyrin **1** (13.8 mg, 10.1%) as a dark red solid. Mp > 200.0ºC; δH($CDCl_3$) −3.77 (2H, br s), 1.28 (12H, m), 2.31 (8H, m), 3.60 (12H, m), 4.03 (8H, m), 10.04 (3H, br s) and 10.05 (1H, s); δC($CDCl_3$) 11.8, 14.5, 26.3, 28.5, 96.4, 97.0, 135.9, 140.8, 147.4 and 147.8; HRMS (EI) *m/z* 534.3730 (534.3717 calcd for $C_{36}H_{46}N_4$ $[M]^+$).

Synthesis of porphyrin ***2***

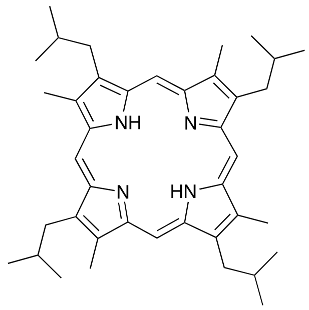

Porphyrin **2** was synthesized from pyrrole **f2** (249.8 mg, 0.810 mmol) according to the procedure described for compound **1**. Isolation of the crude product delivered 12.8 mg (10.7%) as a dark red solid. Mp > 200.0ºC; δH($CDCl_3$) −3.72 (2H, br s), 1.30 (24H, m), 2.70 (4H, m), 3.62 (12H, br s), 3.93 (8H, br t, J = 6.2 Hz), 3.96 (2H, d, J = 3.6 Hz), 10.04 (1H, s), 10.09 (1H, s) and 10.56 (2H, br s); δC($CDCl_3$) 12.5, 23.5, 32.1, 36.1, 98.5, 98.6, 136.7, 140.3, 142.2 and 142.4; HRMS (EI) *m/z* 590.4342 (590.4343 calcd for $C_{40}H_{54}N_4$ $[M]^+$).
